# Cytokines and Oxidative Stress Status Following a Handball Game in Elite Male Players

**DOI:** 10.1155/2011/804873

**Published:** 2011-09-14

**Authors:** Douglas Popp Marin, Rita de Cassia Macedo dos Santos, Anaysa Paola Bolin, Beatriz Alves Guerra, Elaine Hatanaka, Rosemari Otton

**Affiliations:** ^1^Postgraduate Program-Health Sciences, Cruzeiro do Sul University, 03342-000, São Paulo, SP, Brazil; ^2^Metodista University of Sao Paulo, 09640-000 Sao Bernardo do Campo, SP, Brazil; ^3^Postgraduate Program-Human Movement Sciences, Institute of Physical Activity and Sport Sciences, Cruzeiro do Sul University, 01506-000, São Paulo, SP, Brazil; ^4^Laboratório de Fisiologia Celular, Universidade Cruzeiro do Sul, Av. Regente Feijó, 1295, 03342000 São Paulo, SP, Brazil

## Abstract

*Background*. Handball is considered an intermittent sport that places an important stress on a player's aerobic and anaerobic metabolism. However, the oxidative stress responses following a handball game remain unknown. We investigated the responses of plasma and erythrocyte antioxidant system and oxidative stress biomarkers following a single handball game. *Methods*. Fourteen male elite Brazilian handball athletes were recruited in the present study. Blood samples were taken before, immediately, and 24 hours after the game. *Results*. After the game and during 24 hours of recovery, the concentration of all oxidative stress indices changed significantly in a way indicating increased oxidative stress in the blood (thiol groups and reduced glutathione decreased, whereas TBARS and plasma antioxidant capacity was increased) as well as in erythrocyte (increased levels of TBARS and protein carbonyls). Erythrocyte antioxidant enzyme activities were also significantly changed by handball. Muscle damage indices (creatine kinase and lactate dehydrogenase) increased significantly after exercise. In addition, IL-6 increased after the game, whereas TNF-**α** decreased during recovery. *Conclusion*. This study demonstrates that a single handball game in elite athletes induces a marked state of oxidative stress evidenced by the oxidative modification in plasma and erythrocyte macromolecules, as well as by changes in the enzymatic and nonenzymatic antioxidant system.

## 1. Introduction

Sport training involves repeated bouts of exercise and high volume of physically demanding practice sessions and competitive games, which may lead to decline on performance, oxidative stress, and inflammation [1, 2]. In fact, an exercise stimulus has been well recognized to induce the production of reactive oxygen and nitrogen species (RONS) [2, 3]. In spite of RONS having an essential role as signalling molecules in several cellular pathways, the imbalance in the pro- and antioxidant status can lead to detrimental effect on cellular loss of redox homeostasis and oxidative damage in lipids, proteins, and DNA [3]. In addition, an excessive amount of RONS plays a detrimental role in exercise performance through shifting contractile function or muscle acute fatigue [4]. 

Regular exercise training has been associated with upregulation of the antioxidant system capacity to cope with the increase of RONS production. For this reason, pro-oxidant response of elite athletes to an acute exercise stimulus can be usually blunted [3]. Most of the studies regarding exercise-induced oxidative stress were carried out with exercise protocols including typical aerobic (running and cycling) [3, 5] and anaerobic (resistance training and sprints) exercise [6–8]. Different types of exercise may induce varying levels of RONS and affect plasma oxidative stress in a specific way. For example, in response to muscle-damaging exercise, indices of plasma oxidative stress may persist for days, contrasting with the return to the rest values a few hours after an acute non-muscle-damaging exercise protocol [9, 10]. Nikolaidis et al. [8] demonstrated that a muscle-damaging eccentric protocol produced a large increase in plasma oxidative stress parameters until 3 to 4 days after exercise. When compared with continuous exercise, intermittent exercises such as sporting games (soccer, basketball, and handball) involve both aerobic and anaerobic metabolism and have been received few attention in the literature [11, 12]. The physiological load of intermittent exercise, such as handball games, differs from a continuous steady-state exercise [13]. Handball is considered a demanding exercise mode that places important stress on a player's aerobic metabolism. In addition, handball game involves a large number of anaerobic actions such as body-contact, repeated accelerations, sprints, jumps, throwing, blocking, pushing, and rapid changes in moving directions [14, 15]. Studies in elite handball players have focused only on performance parameters like maximal muscle strength, sprint time, and jump height in response to a tournament [14] or a simulated handball match [16]. 

During a competitive handball season, the demand for playing 2 games per week elevates the stress imposed to the athletes, thereby increasing the injury risk and performance decline due to fatigue and muscle damage. Increased oxidative stress caused by training and games may compromise the exercise performance of athletes [1, 17] throughout the competitive season and particularly during the most intensive game's schedule. Recent evidences suggest that increase of exercise-induced oxidative damage was associated with an overtraining state [1]. Considering the physiological and physical demands of handball game, it is reasonable to hypothesize that handball players may be exposed to a condition of oxidative stress. Thus, the purpose of the present study was to investigate the responses of antioxidant system and oxidative stress biomarkers in plasma and erythrocytes following a single handball game.

## 2. Material and Methods

### 2.1. Participants

Fourteen male elite Brazilian handball athletes were recruited into the study (Table 1). They were informed about the experimental procedures and possible discomforts associated with the study before giving their written informed consent to participate. The experimental procedures and protocol conformed to the principles of the Declaration of Helsinki were approved by the Human Ethics Committee of the Cruzeiro do Sul University.

### 2.2. Experimental Design

**Figure 1:**
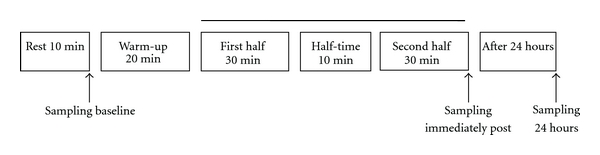
Experimental design.

The standard friendly handball match was conducted in the middle of the handball season, when the athletes are accustomed to playing games. The match consisted of 2 × 30 minutes halves with 10-minute interval at half-time and a 1 minute break between each half. On the match day, players came to the game gymnasium 2 hours before the match start. Blood samples were collected from each athlete: pre-exercise (Baseline), immediately at the end of the second half (Postgame), and 24 hours after the game (24 h) (Figure 1). All athletes had been asked to abstain at least 4 days from games and 2 days from handball training. 

### 2.3. Blood Collection and Analysis

At each time point, blood samples were collected from antecubital vein into 10 mL vacutainer tubes (Vacutainer, Becton Dickinson, USA) containing 0.1 mM ethylenediaminetetraacetic acid (EDTA) solution and immediately processed. Blood was centrifuged at 1,200 rpm for 10 minutes at 4°C to obtain plasma. Erythrocytes were collected from whole blood. Plasma and erythrocytes aliquots (100 *μ*L) were stored at −80°C (Revco, Ashville, NC, USA) until analyzed for the dependent variables. Upon use, the plasma was diluted 1 : 100 with 0.1 mM phosphate buffer, pH 7.4, and used for measurement of the following parameters: oxidative damage, total antioxidant activity (FRAP assay), reduced glutathione (GSH), oxidized glutathione (GSSG) and reduced glutathione/oxidized glutathione ratio (GSH/GSSG), creatine kinase, lactate dehydrogenase and cytokine release. 

Erythrocytes were collected from whole blood after the plasma had been removed. Aliquots of erythrocytes (100 *μ*L) were stored at −80°C until the evaluation of oxidative damages and enzymes activities: total superoxide dismutase, catalase, glucose-6-phosphate dehydrogenase, glutathione peroxidase, and glutathione reductase. All measurements of the present study were carried out in triplicate, and sensitivity of the methods is indicated in the appropriate section. Adequate quality controls were assayed to ensure the quality of the samples. 

### 2.4. Preparation of Homogenates for Measurement of Antioxidant Enzymes and Oxidative Damage

Antioxidant enzymes and oxidative damages were carried out in an aliquot of the erythrocytes (100 *μ*L) hemolyzed by adding four volumes (400 *μ*L) of ice-cold deionized water to yield a hemolysate. Afterwards, erythrocytes were centrifuged for 10 min, 10,000 ×g at 4°C and the supernatant was collected. An additional dilution was carried out producing a final dilution of 1 : 500 using assay-specific extraction solution buffer. After centrifugation (10,000 ×g, at 4°C, 10 min), a clear supernatant was collected and used for further analysis. 

### 2.5. Oxidative Damage (TBARS Assay, Thiol and Carbonyl Group)

Oxidative damages are good strategies to indirectly evaluate RONS production since biomolecules such as lipids and proteins can be easily injured by free radicals. The measurement of thiobarbituric acid reactive substances (TBARS) assay was described by Fraga [18] through the formation of a colored adduct after the stoichiometric reaction between thiobarbituric acid and several lipid-derived aldehydes, including malondialdehyde. The absorbance at 535 nm was measured after the mixture reaches room temperature, and the TBARS content was estimated by a standard curve of 0–10 *μ*M 1,1,3,3-tetraethoxypropane. Thiol and carbonyl groups were evaluated as biomarkers of amino acid oxidation in total protein fractions of plasma and erythrocytes. Diluted erythrocytes and plasma were precipitated with 20% trichloroacetic acid solution in ice. Reduced thiol groups were detected by the formation of colored adducts after reaction with 4 mM 5.5′-dithio-*bis *(2-nitrobenzoic acid) solution (DTNB). The absorbance of DTNB-treated samples at 412 nm was calculated using GSH as a standard (0–100 *μ*M) [19]. The same procedure was used to estimate protein carbonyls. The protein carbonyls were identified by the hydrazones formed with 10 mM dinitrophenylhydrazine in 0.25 M HCl. Absorbance of the peak detected within the range of 340–380 nm was measured, and the carbonyl group concentration was calculated based on the molar coefficient of *ε* = 2.2 × 10^4^ M^−1^ cm^−1^ [20] using the bovine serum albumin (BSA) as a standard (0–1000 *μ*g/mL).

### 2.6. Erythrocytes Enzyme Activities

Total superoxide dismutase, catalase, glucose-6-phosphate dehydrogenase, glutathione peroxidase, and glutathione reductase activities were determined in erythrocytes using a spectrophotometer (Amersham Biosciences, UK). Catalase activity was measured as described by Aebi [21] based on the direct decomposition of hydrogen peroxide (H_2_O_2_). Total superoxide dismutase activity was measured using the method described by Ewing and Janero [22] which involves the reduction of $\begin{array}{c} {{\text{O}}_{2}}^{●-} \end{array}$ radicals by nitroblue tetrazolium following a linear first-order kinetic during 3 min. Glutathione peroxidase [23] and glutathione reductase [24] activities were measured based on the oxidation of *β*-NADPH in the presence of *tert*-butyl hydroperoxide used as substrate. The activity of glucose-6-phosphate dehydrogenase was determined as described in our previous study [25].

### 2.7. Total Antioxidant Activity (FRAP Assay)

Determination of ferric-reducing ability of plasma (FRAP assay) was based on a single electron transfer reaction between plasma antioxidants and Fe_3_ utilized as oxidant. The stock solution included 0.1 M acetate buffer, pH 3.6, 10 mM TPTZ (2,4,6-tripyridyl-s-treazine) solution in 40 mM HCl, and 20 mM FeCl_3_·6H_2_O solution. FRAP solution reagent (200 *μ*L) prepared freshly was mixed with 40 *μ*L of distilled water and 10 *μ*L of plasma sample. The change absorbance of the Fe_2_ complex, due to the action of an antioxidant in the plasma, was measured at 593 nm by spectrophotometer (Tecan, Salzburg, Austria). The standard curve was linear between 0 and 100 *μ*M FeSO_4_.

### 2.8. GSH and GSSG Determination

Whole blood was used for determination of glutathione status, using the method described by Rahman [26]. Both total GSH and GSSG were analyzed using 5,5′-diothiobis-2 nitrobenzoic acid to combine with reduced glutathione to form 5-thio-2-nitrobenzoic acid. The GSH/GSSG concentration was calculated from a standard curve prepared with pure GSH/GSSG standards (0–50 nM) and was expressed as nM of GSH and GSSG.

### 2.9. Muscle Damage Markers

Plasma creatine kinase and lactate dehydrogenase levels were analyzed using a detecting kit Bioliquid Creatine kinase-NAC and lactate dehydrogenase obtained from Laborclin (Paraná, Brazil). The lactate dehydrogenase method is based on reduction of pyruvate with NADH, resulting in lactate and NAD+. The catalytic concentration is determined by the speed of the decomposition of NADH, measured by the decrease in absorbance at 340 nm. Creatine kinase was assayed using an enzymatic method based on the rate of NADPH formation that absorbs at 340 nm. All samples were compared to known standards on a spectrophotometer (Amersham-Biosciences Ultrospec 3100-pro).

### 2.10. Cytokines Release

Cytokines IL-6 and TNF-alpha were assayed in plasma with ELISA kits according to the manufacturer's instructions (Quantikine, R&D System, Minneapolis, Minn, USA). The lower limits of detection for the ELISA analyses were as follows: 1.56 pg/ml for IL-6 and 1.5 pg/ml for TNF.

### 2.11. Protein Determination

The total protein content of plasma was measured by the method of Bradford [27], using BSA as standard.

### 2.12. Statistical Analyses

Data are presented as means ± standard error of the mean. Statistical inferences were made by the one-way analysis of variance for repeated measures, and a Tukey's *post hoc* test was used (INStat; Graph Pad Software, San Diego, Calif, USA). *P* values below 0.05 were considered statistically significant. In addition, percentage changes and effect sizes (ES) for the difference between pre- and postgame means were calculated. ES was calculated as the difference between two sample means, divided by the average standard deviation of the two samples. The scale for determining the magnitude of ES for elite athletes was interpreted according to [28] as follows: 0.25 = trivial, 0.25–0.5 = small, 0.5–1.0 = moderate, and >1.0 = large. 

## 3. Results

### 3.1. Oxidative Damages in Plasma and Erythrocytes

In the present study, we initially evaluated the effect of single handball game on oxidative stress in trained athletes by measuring the levels of lipid peroxidation and protein oxidation in both plasma and erythrocytes as shown in Table 2. In the plasma, there was an increase in TBARS levels (62% ES = 0.99) in parallel to a marked decrease in thiol groups (59% ES = 2.22), immediately postgame. Plasma TBARS levels remained elevated during recovery and reached its highest level at 24 hours (107% ES = 1.49), while the levels of thiol groups returned to baseline at 24 hours. No alterations in plasma carbonyls groups after exercise and recovery were observed. Erythrocyte TBARS levels were significantly increased 24 h in response to handball game (153% ES = 2.63) in comparison to baseline, whereas no significant difference was observed in erythrocyte thiol groups. On the other hand, erythrocyte carbonyls groups increased significantly immediately postgame (40% ES = 2.29) and after 24 h of recovery (47% ES = 3.19). Our findings indicated a significant increase of oxidative biomarkers on plasma and erythrocytes in handball athletes after the game.

### 3.2. FRAP Level and GSH/GSSG Ratio

**Figure 2:**
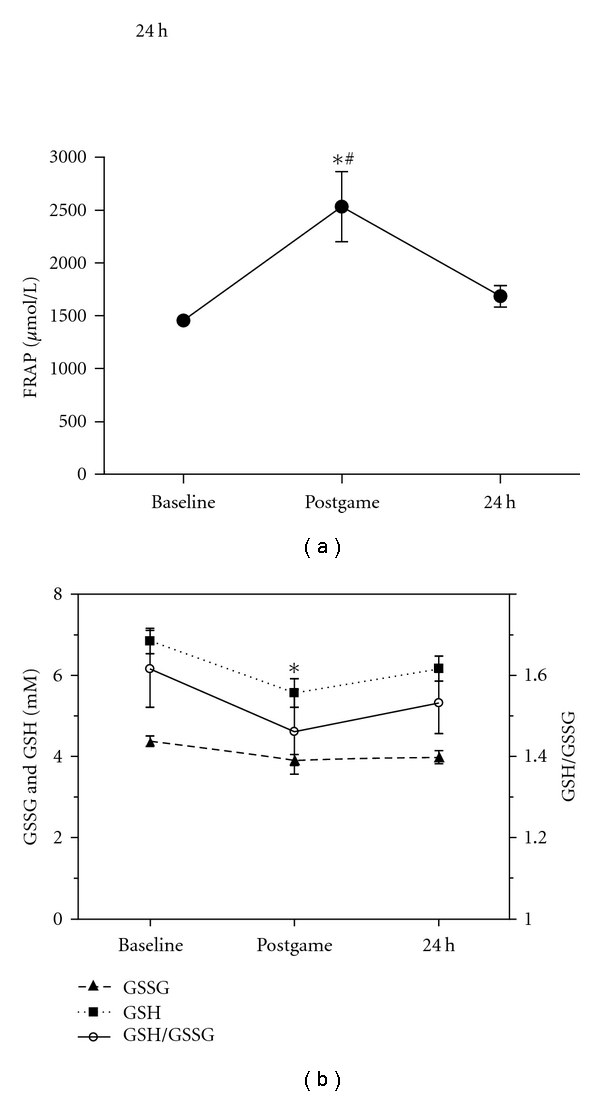
FRAP level (a) and GSH/ GSSG ratio (b) in plasma of athletes following a handball game. Results are presented as mean ± SEM. *Indicates significant differences in respect to baseline values (*P < *0.05), and # indicates significant differences between postmatch and 24 h after (*P < *0.05).

Antioxidant activity of the plasma was assessed by the FRAP assay. The FRAP level was significantly increased immediately postgame (74% ES = 1.58) and returned to baseline levels at 24 hours (Figure 2(a)). These results point out to a marked mobilization of antioxidants defences during exercise. The plasma GSH and GSH/GSSG ration is considered an important marker of oxidative stress. GSH concentration significantly decreased postgame (18% ES = 0.71) and returned toward the baseline during recovery (Figure 2(b)). No changes were observed in GSSG concentration and GSH/GSSG ratio after the game. 

### 3.3. Creatine kinase and Lactate Dehydrogenase

**Figure 3:**
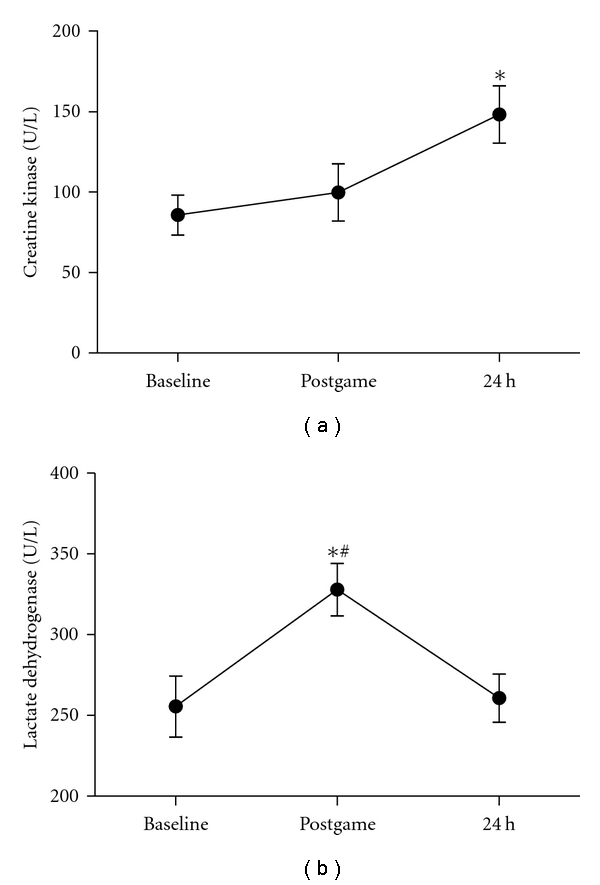
Creatine kinase (a) and lactate dehydrogenase (b) activity in plasma of athletes following a handball game. Results are presented as mean ± SEM. *Indicates significant differences in respect to baseline values (*P < *0.05), and # indicates significant differences between postmatch and 24 h after (*P < *0.05).

Creatine kinase and lactate dehydrogenase have been used as an indirect marker of muscle damage. Interestingly, plasma levels of creatine kinase (Figure 3(a)) increased significantly at 24 hours as compared to baseline levels (72% ES = 1.52). Plasma lactate dehydrogenase levels increased significantly (28% ES = 1.36) immediately postgame and returned to baseline levels after recovery (Figure 3(b)). These results were unexpected since the subjects engaged in the present study were highly trained athletes and well familiarized to competitive games. 

### 3.4. Cytokine Release

**Figure 4:**
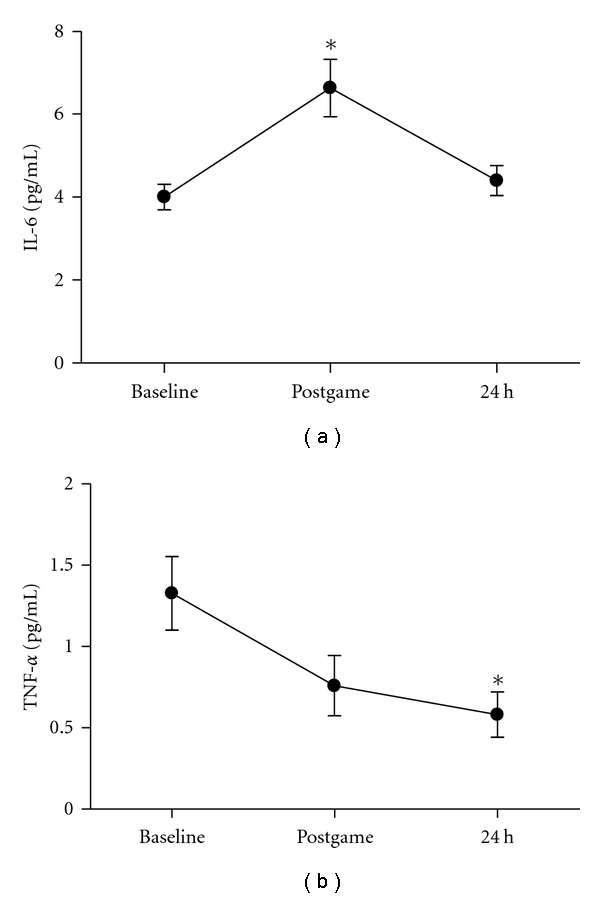
IL-6 (a) and TNF-alpha (b) in plasma of athletes following a handball game. Results are presented as mean + SEM. *Indicates significant differences in respect to baseline values (*P < *0.05).

Consistent with previous studies, plasma IL-6 levels increased significantly only immediately postgame (66% ES = 2.09) and normalized at 24 hours (Figure 4(a)). On the other hand, the other unexpected result of our study was the significantly reduction of TNF-*α* (56% ES = 0.67) after 24 hours of recovery (Figure 4(b)).

### 3.5. Erythrocyte Enzyme Activities

Maximal antioxidant enzyme activities are used as biomarkers of oxidative stress and for the monitoring of adaptative response to exercise (Table 3). The handball game induced a significantly increase in total erythrocyte superoxide dismutase activity at postgame (166% ES = 3.53) and at 24 h recovery (248% ES = 3.66), supporting the idea that chronic exercise training may improve the response of this enzyme. Erythrocyte catalase was significantly decreased immediately after the game (−36% ES = 0.66) and 24 h of recovery (−57% ES = 1.30) in comparison to baseline. In the same way, erythrocyte glutathione reductase was significantly decreased after the game (−46% ES = 0.93) and 24 h of recovery when compared with baseline values (−53% ES = 1.12). Glutathione peroxidase and glucose-6-phosphate dehydrogenase activity were not altered immediately and at 24 h after the handball game. Regarding our experimental design, we observed a significant increase in the antioxidant enzymes activities after a handball game to combat the excessive RONS production during exercise and recovery.

## 4. Discussion

The present investigation shows that a friendly handball match by Brazilian elite handball players alters significantly the response of the oxidative stress biomarkers, the antioxidant capacity, and the indices of muscle damage in plasma and/or erythrocytes, both immediately and 24 hours after the game. During a handball game, about 90% of the energy released must be aerobically driven and athletes run about 4–6 km at a mean intensity to 80–90% of maximal heart rate [29]. Also, a level of 94% VO_2max_ has been achieved during a specific handball exercise [13]. At this condition, an estimated 1–5% of the total oxygen consumption results in the formation of superoxide anion [30], although a more recent study reports much lower values [31]. Given the high intensity of handball, it is not surprising that the biomarkers of oxidative stress were significantly increased following the game. Further mechanisms can contribute to RONS generation during exercise in addition to electron leakage from the mitochondrial electron chain, such as xanthine oxidase production, catecholamine and oxyhemoglobin auto-oxidation, and phagocytic respiratory burst activity [4, 5]. The proton accumulation due to lactic acidosis, which has been demonstrated in vitro to be a potent prooxidant factor [32], can also contribute to RONS formation during the handball game. In addition, blood interacts with all organs and tissues and, consequently, with many possible sources of RONS [33].

Increase in RONS production during exercise can rapidly react with lipids in plasma and cell membranes, proteins, and other cell components [3, 5]. The imbalance between antioxidant capacity and oxidant production induced by handball is supported by increased oxidative damage in plasma and erythrocytes. The handball game induced a significant decrease in plasma thiol groups, as evidenced by increased formation of disulfide bridges in proteins. An important feature of most thiol is that they can act as reducing agents. RONS have a strong tendency to gain electrons to other species, consequently, in the case of an oxidant-thiol interaction; the oxidant is neutralized to a relatively lesser toxic by-product. 

Erythrocytes are susceptible to oxidative damage at rest and during exercise as a result of the high content of oxygen, heme iron, and polyunsaturated fatty acids. Although plasma protein carbonyls did not change after the game, we found a significant increase in the erythrocyte protein carbonyls after the game and during 24 h of recovery. Our findings are in accordance with Senturk et al. [34] who reported a significant increase in protein carbonyls after exhaustive cycling exercise in trained subjects. In the same way, increased erythrocyte and plasma TBARS levels reveal a high susceptibility of unsaturated fatty acids to oxidative modification during increased RONS production [33]. Recent studies have published similar results on blood oxidative stress biomarkers in response to a soccer game [11, 12] and indoor climbing [35]. Lipid peroxidation results in changes of membrane integrity and disruption of cellular processes and reduces the capacity of the cell to maintain the ion gradient [4]. Regarding exercise performance, lipid peroxidation has been associated with tissue inflammation, muscle fatigue, and impaired recovery following high-intensity exercise [36]. Several studies have reported an increase in TBARS following both maximal and submaximal non-muscle-damaging exercise in humans [3], with values typically returning to baseline within one hour after exercise [37]. However, in accordance with previous studies, we observed a large (ES = 1.49) 24-hours postexercise increase in plasma and erythrocyte (ES = 2.63) TBARS levels [1, 38]. This response of TBARS can persist until 1 to 4 days after muscle-damaging exercise [39]. In this respect, we observed a marked elevation of lactate dehydrogenase (ES = 1.36) and creatine kinase (ES = 1.52) levels providing an indirect evidence of muscle damage after a single handball game. The lactate dehydrogenase and creatine kinase efflux from muscle may be attributed to the increased permeability of plasma membrane and/or intramuscular vasculature [40]. Peak levels of creatine kinase occurred 24 hours following the game similarly as reported after a soccer match [11]. The increased damage of muscle membrane in the days after exercise can potentially lead to increased concentration of unsaturated fatty acids in the blood, which may be one of the mechanisms through which muscle-damaging exercise increased plasma lipid peroxidation for days after exercise [39]. 

Although not measured in this study, another mechanism involved in exercise-induced RONS production related to muscle damaging is the activation and infiltration of phagocytes into the injured muscle during recovery after exercise [35, 41]. Activated neutrophils are a major source of oxidants, because they use a variety of RONS, including superoxide anion, hydrogen peroxide, and hypochlorous acid to destroy damaged tissue leading to postexercise inflammation, removal of traumatized tissue, and muscle repair [39]. 

RONS production may be involved in the regulation of redox-sensitive transcription factors that mediate the expression of inflammatory mediators such as cytokines, chemokines, and adhesion molecules [42]. Cytokines are involved in the control of the immune and acute-phase response, inflammatory reactions, and tissue repair process [12, 43]. IL-6 concentration increased (ES = 2.09) only immediately after the game. Although IL-6 is produced early in inflammation, it cannot be regarded as a typically proinflammatory cytokine. IL-6 values peak appears at the end of an intense bout of exercise, or within a few hours, and then decrease rapidly to baseline levels [44]. The magnitude of the response of plasma-IL-6 is related to exercise duration, intensity, and muscle mass recruited during activity [43]. Muscle damage is not required in order to increase plasma IL-6 during exercise [45]. The skeletal muscle can produce IL-6 during exercise in the absence of observable markers of inflammation and in a TNF-*α*-independent way, linking IL-6 to metabolism rather than inflammation [45]. Previous researchers have demonstrated that IL-6 mRNA and transcriptional rate of IL-6 gene are upregulated in contracting skeletal muscle fibres and then released from working muscle into the circulation [44]. In response to exercise, IL-6 acts in an autocrine and paracrine manner to maintain glucose homeostasis, induce lipolysis and fat oxidation, exert an anti-inflammatory effect by suppression of TNF-*α* production, and also stimulate muscle repair by the proliferation of satellite cells and differentiation of myoblasts [46]. 

GSH/GSSG ratio has been studied following aerobic and anaerobic exercise as a sensitive marker of oxidative stress and has been found to be decreased after exercise [9]. Together with thiol groups, GSH plays a multifunctional role in protecting biomolecules from oxidative damage during exercise. Reduced concentration of GSH immediately after the game is in accordance with increases in plasma TBARS, thiol groups, and erythrocytes protein carbonyls at the same sampling time and, therefore, indicates increased RONS production. Similar to our findings, Andersson and coworkers [47] reported a significant decreased in GSH after a soccer game in well-trained female soccer players. The decrease in GSH concentration may be explained by its consumption either to regenerate ascorbic acid and alpha-tocopherol or to directly scavenge RONS. In the present study, we found that FRAP robustly increased (ES = 1.58) after the handball game as have been published by previous studies [11, 12]. This increase suggests an acute compensatory response of the body's antioxidant defences against the RONS production. Antioxidant capacity elevated after exercise seems to be partially supported by an increase in uric acid [11] and plasma catalase activity [48]. Nonetheless, the mobilization of FRAP observed during and immediately after exercise was not sufficient to avoid oxidative modification in biomolecules of the blood. In this sense, there is now compelling evidence that erythrocytes contribute to the maintenance of circulatory antioxidant levels [33]. Conflicting results have been reported about erythrocyte antioxidant enzyme activities in response to acute exercise. The observed changes in erythrocyte antioxidant enzymes following a handball game and 24 h of recovery are quite different from those reported after mountain cycling race [49] or following maximal and submaximal exercise test [50]. Our results demonstrated a significant increase of total superoxide dismutase activity both after the game and during the recovery, whereas no changes have been shown after duathlon competition [51] or prolonged exercise test [50]. Since erythrocytes cannot synthesize proteins, the increase in the enzyme activity could be attributed to covalent modification of proteins or other protein interactions [51]. In addition, long-term effects of oxidative stress in handball athletes are reflected in the induction of adaptation, especially in the increase of superoxide dismutase activity at rest and in response to an acute intense exercise stimulus [52]. Previous studies have reported increases [49], decreases [51], or even no changes [53] in erythrocyte catalase and glutathione peroxidase activity after exercise. We demonstrated that catalase activity was decreased after both handball game and recovery, while no significant difference was observed in glutathione peroxidase activity. These changes are in agreement with the antioxidant defences being overwhelmed by handball game. Previous studies also reported that glutathione reductase activity may increase [49] or not change [50] after exhaustive exercise. In the present study, the handball game significantly decreased glutathione reductase activity throughout the recovery period. Tauler et al. [54] pointed out that the glutathione reductase activity was inhibited possibly by an increase in hydrogen peroxide concentration. Along this line, catalase is also inhibited by high concentrations of hydrogen peroxide [21], suggesting a depletion of the enzymatic antioxidant pool. 

Chronic exercise training has been associated with a training-induced adaptive response against oxidative stress [5]. Since that subjects employed in the current investigation were elite athletes and well familiarized to competitive games, it may be speculated that the oxidative stress following a handball game may not be prevented. In this respect, the antioxidant defence system seems to control levels of RONS, rather than eliminating them completely [4]. This seems logical, as RONS plays numerous physiological roles in cellular signalling, muscle contraction, glycogen repletion, and enzyme activity [5].

Many studies have suggested the administration of dietary antioxidant before and after exercise bouts in an attempt to attenuate any potential increase in oxidative stress, although outcomes appear to be influenced not only by level of exercise, but also by the amount, duration, and the type of supplement antioxidant [4, 55]. As evidenced by previous studies, the administration of an antioxidant supplement before the handball match would not improve performance but rather provide protection against the negative consequences of free radicals produced during the game in competitive athletes [55, 56]. 

 In conclusion, this study demonstrates that a single handball game in elite athletes induced a state marked by oxidative stress evidenced by the oxidative modification in plasma and erythrocyte macromolecules and by changes in the enzymatic and nonenzymatic antioxidant system. Increased oxidative stress caused by training and games may compromise the exercise performance of athletes such as strength and power over a competitive season and particularly during the most intensive game's schedule. Further studies are suggested to evaluate the oxidative stress and inflammatory markers throughout the sport season (club training and matches) in both male and female athletes. In addition, the impact of antioxidant supplementation on the recovery processes after a single handball game is still to be determined.

## Figures and Tables

**Table 1:** Antropometric characteristics of the athletes.

	Mean ± SD
Age (yr)	25 ± 4.5
Height (cm)	187.7 ± 6.6
Body mass (kg)	95.3 ± 9.8
Body fat (%)	13.8 ± 3.1
Maximal oxygen consumption (mL/kg/min)	51.9 ± 2.1
Training per week (h)	15 ± 0.5
Handball practice (yr)	11.4 ± 3.1

**Table 2:** Plasma and erythrocyte TBARS levels, protein carbonyl, and thiol content of handball athletes. Results are presented as mean ± SEM. *Indicates significant differences in respect to baseline values (*P < *0.05).

	Baseline	Postgame	24 h
*TBARS *			
Plasma (*μ*mol MDA/mL)	0.11 ± 0.02	0.18 ± 0.01*	0.24 ± 0.02*
Erythrocytes (*μ*mol MDA/mg of protein)	668.80 ± 50.87	595 ± 62.42	1687 ± 184.20*

*Protein Carbonyls *			
Plasma (protein carbonyls/mL)	16.16 ± 0.37	14.73 ± 0.58	14.64 ± 0.27
Erythrocytes (protein carbonyls/mg of protein)	42.77 ± 0.92	59.95 ± 2.94*	62.78 ± 2.30*

*Thiols *			
Plasma (mmol thiols/mL)	20.40 ± 2.09	8.23 ± 1.01*	16.67 ± 1.35
Erythrocytes (mmol thiols/mg of protein)	1.57 ± 0.29	1.22 ± 0.22	1.05 ± 0.21

**Table 3:** Erythrocyte antioxidant enzyme activities of handball athletes. Results are presented as mean ± SEM. (*) Indicates significant differences in respect to baseline values (*P < *0.05), and (^#^) indicates significant differences between postmatch and 24 h after (*P < *0.05).

	Baseline	Postgame	24 h
Total superoxide dismutase (mU/mg)	0.11 ± 0.01	0.31 ± 0.01*	0.41 ± 0.03^∗#^
Catalase (*μ*mol/mg)	4.99 ± 0.59	3.20 ± 0.51*	2.12 ± 0.33*
Glutathione peroxidase (mU/mg)	10.56 ± 2.54	9.25 ± 1.79	9.61 ± 1.53
Glutathione reductase (mU/mg)	2.29 ± 0.04	1.23 ± 0.18*	1.05 ± 0.20*
Glucose-6-phosphate dehydrogenase (mU/mg)	445.80 ± 138.19	473.60 ± 25.16	477.20 ± 20.61
